# Adeno-associated virus vector intraperitoneal injection induces colonic mucosa and submucosa transduction and alters the diversity and composition of the faecal microbiota in rats

**DOI:** 10.3389/fcimb.2022.1028380

**Published:** 2022-12-22

**Authors:** Li-Tian Ma, Jing-Xuan Lian, Yang Bai, Meng-Juan Shang, Zhe-Zhe Zhang, Fei-Fei Wu, Jing Chen, Xian-Bo Meng, Jin Zheng, Tian Li, Yun-Qing Li, Jing-Jie Wang

**Affiliations:** ^1^ Department of Gastroenterology, Tangdu Hospital, Air Force Medical University, Xi’an, China; ^2^ Department of Traditional Chinese Medicine, Tangdu Hospital, Air Force Medical University, Xi’an, China; ^3^ Department of Endocrinology, Xijing Hospital, Air Force Medical University, Xi’an, China; ^4^ Department of Neurosurgery, General Hospital of Northern Theater Command, Shenyang, China; ^5^ Department of Radiation Biology, Faculty of Preventive Medicine, Fourth Military Medical University, Xi'an, ShaanXi, China; ^6^ National Demonstration Center for Experimental Preclinical Medicine Education, Air Force Medical University, Xi’an, China; ^7^ Department of Anatomy, Histology and Embryology & K. K. Leung Brain Research Centre, The Fourth Military Medical University, Xi'an, China; ^8^ School of Basic Medicine, Fourth Military Medical University, Xi'an, China; ^9^ Key Laboratory of Brain Science Research and Transformation in Tropical Environment of Hainan Province, Haikou, China; ^10^ Department of Anatomy, College of Basic Medicine, Dali University, Dali, China

**Keywords:** adeno-associated virus vector (AAV), faecal microbiota, diversity, 16S rRNA, gene therapy

## Abstract

**Background:**

Viral vector technology, especially recombinant adeno-associated virus vector (rAAV) technology, has shown great promise in preclinical research for clinical applications. Several studies have confirmed that rAAV can successfully transduce the enteric nervous system (ENS), and rAAV gene therapy has been approved by the Food and Drug Administration (FDA) for the treatment of the early childhood blindness disease Leber congenital amaurosis and spinal muscular atrophy (SMA). However, until now, it has not been possible to determine the effect of AAV9 on intestinal microbiota.

**Methods:**

We examined the efficiency of AAV9-mediated ascending colon, transverse colon and descending colon transduction through intraperitoneal (IP) injection, performed 16S rRNA gene amplicon sequencing and analysed specific faecal microbial signatures following AAV9 IP injection via bioinformatics methods in Sprague–Dawley (SD) rats.

**Results:**

Our results showed (1) efficient transduction of the mucosa and submucosa of the ascending, transverse, and descending colon following AAV9 IP injection; (2) a decreased alpha diversity and an altered overall microbial composition following AAV9 IP injection; (3) significant enrichments in a total of 5 phyla, 10 classes, 13 orders, 15 families, 29 genera, and 230 OTUs following AAV9 IP injection; and (4) AAV9 can significantly upregulate the relative abundance of anaerobic microbiota which is one of the seven high-level phenotypes that BugBase could predict.

**Conclusion:**

In summary, these data show that IP injection of AAV9 can successfully induce the transduction of the colonic mucosa and submucosa and alter the diversity and composition of the faecal microbiota in rats.

## Introduction

Viral vector technology, especially recombinant adeno-associated virus vector (rAAV) technology, has shown great promise in preclinical research for clinical applications. rAAV is more widely used than other viral vectors because it can achieve a high level of transduction within a few weeks that can be maintained throughout the life cycle of the organism (Flotte, 2004). The peripheral nervous system, including the autonomic nervous system (ANS) (Sydney-Smith et al., 2021) and enteric nervous system (ENS), can be transduced following systemic rAAV injection (Chan et al., 2017). Furthermore, the low immunogenicity of rAAV further promotes its application in the biomedical field (Buckinx and Timmermans, 2016). Surprisingly, after unremitting basic research and clinical trials, rAAV gene therapy has been approved by the Food and Drug Administration (FDA) for the treatment of the early childhood blindness diseases Leber congenital amaurosis (Kumaran et al., 2018) and spinal muscular atrophy (SMA) (Hoy, 2019).

Several studies have confirmed that rAAV can successfully transduce the ENS. For example, Benskey et al (Benskey et al., 2015) injected rAAV expressing green fluorescent protein (GFP) directly into the wall of the descending colon in adult rats and found that a single injection of AAV into the colon covered an area of ~47 mm^2^, and rAAV9 primarily transduced neurons, while rAAV6 transduced enteric glia and neurons. In addition to direct injection into the colon wall, intravenous rAAV9 injection also induced ENS transduction within the stomach, small intestine and large intestine in cynomolgus macaques (Gombash et al., 2017). Related research has reached similar conclusions (Buckinx et al., 2016). In addition to ENS transduction, intracolonically (IC)-administered rAAV can also efficiently transduce the colonic mucosa in rats and might have therapeutic potential for ulcerative colitis (UC) (Hao et al., 2012; Zheng et al., 2017). Various routes are used to deliver rAAV, such as oral administration, intraperitoneal (IP) injection, superior mesenteric artery injection, enema, and the colon wall injection mentioned above. Polyak et al (Polyak et al., 2008) revealed the tropism and efficiency of gene delivery to intestinal epithelial cells using rAAV pseudotypes 2/1, 2/2 and 2/5. Although rAAV use has been approved in the treatment of certain diseases and is extremely important in the transduction of the central nervous system (CNS), the use of rAAV in the delivery of genes to the gastrointestinal (GI) system has not been fully characterized. One of the purposes of the present study was to examine the efficiency of AAV9-mediated ascending colon, transverse colon and descending colon transduction through IP injection. Furthermore, we explored the effect of AAV9 on the faecal microbiota in rats by IP injection.

There is no doubt that AAV can successfully infect ENS and intestinal epithelial cells; to date, little is known about the effect of rAAV on the intestinal microbiota after transduction. In contrast to the CNS, the GI tract of mammals is a complicated collection of microorganisms, including viruses, bacteria and fungi (Qin et al., 2010; Yu et al., 2021). Dysbiosis could induce GI and neurological damage related to complex diseases and then aggravate the patient’s discomfort (Yu et al., 2021). For instance, some toxic metabolites of intestinal microbiota, such as phenol, ammonia, para-cresol, hydrogen sulfide and amines, can lead to damage to the intestinal mucosal epithelial barrier and further lead to cancer (Cotillard et al., 2013; Yu et al., 2021). Moreover, the gut microbiota can also influence the CNS through the gut-brain axis. For example, damage to dopaminergic neurons in the substantia nigra of the CNS and the formation of Lewy bodies are the main pathological changes that occur in Parkinson’s disease (PD) (Chen et al., 2021). Similarly, these pathological changes are also found in the intestine and are accompanied by dysbiosis (Chen et al., 2021).

In addition, in the first long-term (12-15 years) follow-up of human intravascular delivery of AAV for gene transfer, it was found that 1 subject died from myocardial infarction related to underlying atherosclerotic cardiovascular disease (George et al., 2020). Existing research has revealed that intestinal microbiota play an important role in human cardiovascular diseases (Tang et al., 2017; Zununi Vahed et al., 2018). In particular, bacterial DNA can be detected in the atherosclerotic plaques of individuals with cardiovascular disease, and in each individual, the bacteria found in the intestine can also be observed in the atherosclerotic plaques (Ott et al., 2006; Koren et al., 2011; Tang et al., 2017). Until now, it has not been possible to determine the effect of AAV9 on intestinal microbiota. Therefore, before AAV gene therapy is widely used, especially through oral IP injection, superior mesenteric artery injection, enema and colon wall injection, we must evaluate its impact on intestinal and faecal microbiota. The administration of a small amount of rAAV into the CNS may have little impact on the intestinal microbiota; however, the gut-brain axis is a bidirectional communication system (Leclair-Visonneau et al., 2020). O’Donovan and colleagues showed that bilateral nigral injection of AAV-α-synuclein in rats was accompanied by changes in the ENS and the gut microbiota, whereas there was no apparent brain-to-gut spread of human injected α-synuclein (Leclair-Visonneau et al., 2020; O’Donovan et al., 2020). The possible explanation for this phenomenon is that the gut-brain axis is a bidirectional communication system, but we cannot rule out that rAAV itself leads to changes in the microbiota. Despite these discoveries, to date, little is known about the effect of AAV9 IP injection on faecal microorganisms. Therefore, we used 16S rRNA gene amplicon sequencing and analysed the specific faecal microbial signature following AAV9 IP injection *via* a bioinformatics method in *Sprague–Dawley (SD)* rats. Rats were chosen for the study because previous research reported that the gut microbiota profile of SD rats is closer to that of humans than that of mice using 16S rRNA gene amplicon sequencing (Flemer et al., 2017), and further, the data of metagenome sequencing revealed that 97% of the functional pathways in the human gene catalogue are found in the rat gene catalogue (Pan et al., 2018).

## Materials and methods

### Animals

All animal procedures in this research conformed to the Guide for the Care and Use of Laboratory Animals published by the US National Institutes of Health (NIH publication No. 85–23, revised 1996) and were approved by the Institutional Animal Care and Use Committee of the Air Force Medical University (permit number IACUC-20200503). Every measurement was taken to minimize the discomfort of the animals. A total of 18 male *Sprague–Dawley* rats (250 – 280 g, aged 7 - 8 weeks) were maintained on a 12-hour/12-hour light/dark (LD) cycle and in a temperature- and humidity-controlled environment (23°C, 50% humidity) for two weeks prior to experimentation. Eighteen rats were randomly divided into two groups, the control group and the AAV9 group, with 9 rats in each group. To examine the efficiency of AAV9-mediated colon transduction, we transduced the AAV9-CMV-eGFP system (designed and synthesized by Hanbio, Shanghai, China) into 9 rats at a dose of 1 × 10^12^ vg/ml, 200 µl per rat through IP injection. The control group was injected with an equal volume (200 µl) of saline through IP injection. Animals used for tissue collection were euthanized by an overdose of 2% pentobarbital sodium (IP, 100 mg/kg) after injection for 30 days.

### Colon tissue collection

Briefly, the entire colon, from the ileocecal junction to the rectum, was removed from euthanized rats and rinsed in room temperature phosphate buffered saline (PBS). Next, the ascending colon, transverse colon and descending colon regions were separated. Each segment (i.e., the ascending, transverse, and descending segments) of the colon was directly placed into paraformaldehyde (protected from light) for 12 hours of fixation and then transferred into 30% sucrose (protected from light) for 72 hours. Finally, the nonligated intestinal segment was frozen sectioned. A panoramic pathological scanner (PanoVIEW VS200, Olympus, Japan) was then used to observe GFP expression in the mucosa and submucosa.

### 16S rRNA gene amplicon sequencing of faecal microbiota

Fresh faecal samples of the control and AAV9 group rats (all n = 18) were collected in cryogenic vials and kept in liquid nitrogen. DNA concentration and purity were monitored on 2% agarose gels. 16S rRNA gene amplicon sequencing of faecal microbiota was processed and sequenced by the Illumina HiSeq™ platform (Sangon Biotech Co., Ltd. Shanghai, China). Total faecal DNA was extracted by the E.Z.N.A.™ Mag-Bind DNA Kit. 16S rRNA genes of distinct regions (V3-V4) were amplified using specific primers (Nobar_341F 5’-CCTACGGGNGGCWGCAG-3’ and Nobar_806R 5’-GACTACHVGGGTATCTAATCC-3’) with barcodes (Wear et al., 2018). The paired-end (PE) reads obtained by second-generation sequencing were first spliced according to the overlap relationship.

After distinguishing the samples, the sequence quality was controlled and filtered, and then operational taxonomic unit (OTU) clustering (amplicon sequence variant (ASV) denoising) analysis and species taxonomic analysis were performed. Based on the results of OTU clustering (ASV denoising) analysis, multiple diversity index analysis of OTUs (ASV denoising) and sequencing depth detection can be performed. Based on taxonomic information, statistical analysis of community structure can be performed at each classification level. On the basis of the above analyses, a series of in-depth statistical and visual analyses, such as beta-diversity analysis, group test analysis, significance difference tests and function prediction, can be performed on the community composition and phylogenetic information of multiple samples. The databases used included (1) the RDP 16S database (http://rdp.cme.msu.edu/misc/resources.jsp), (2) the Silva (Quast et al., 2013) 16S database (http://www.arb-silva.de/), (3) the NCBI 16S database (http://ncbi.nlm.nih.gov/); and (4) the GTDB database (https://gtdb.ecogenomic.org/). Futhermore, we used the BugBase database (https://bugbase.cs.umn.edu/) to predict the phenotypes of prokaryotic microorganisms in fecal samples, including gram positive, gram negative, biofilm forming, pathogenic, mobile element containing, oxygen utilizing (including aerobic, anaerobic and facultatively anaerobic) and oxidative stress tolerant (Ward et al., 2017).

### Quantification and statistical analysis

The number of GFP-positive cells in whole mounts from at least three animals was counted (the exact *n* is indicated in the Results section). In frozen slices, three 500 µm^2^ areas were randomly selected for counting the number of GFP-positive cells in the mucosa; all submucosal slice areas were selected for counting the number of GFP-positive cells. The average number of GFP-positive cells in the three areas was taken to represent the number of GFP-positive cells in these slices. The numbers of GFP-positive cells were statistically evaluated with one-way analysis of variance (ANOVA) at a *P* = 0.05 significance level by Prism 7.0 software (GraphPad Software, https://www.graphpad.com/). The faecal DNA samples of all rats (total *n* = 18) were sequenced. All 16S rRNA gene amplicon sequencing statistical analyses were performed using R packages (V.2.15.3). Student’s *t* test was used to compare the alpha diversity of the two groups, including community richness (the *Chao* (http://www.mothur.org/wiki/Chao) and *ACE* (http://www.mothur.org/wiki/Ace) indices), community diversity (the *Shannon* (http://www.mothur.org/wiki/Shannon) and *Simpson* (http://www.mothur.org/wiki/Simpson) indices), and community evenness (the *Shannon evenness* index (Pielou’s evenness index, *J*)) and *p* < 0.05 was considered significant. Statistical Analysis of Metagenomic Profiles (STAMP; v2.1.3) was used to compare the species abundances in the two groups, along with Welch’s *t test* (White et al., 2009; Parks et al., 2014; Bunyavanich et al., 2016; Chi et al., 2018; Raghupathi et al., 2018). Then, we used the false detection rate (FDR) to perform multiple test corrections on the *p* value obtained from Welch’s *t test* to obtain the corrected *p* value, and a corrected *p* < 0.05 was considered statistically significant. We applied linear discriminant analysis effect size (LEfSe) analysis to identify taxa or pathways with different abundances between the control group and the AAV9 group. Futhermore, when using the BugBase database to predict the phenotype of prokaryotic microorganisms, the Mann‐Whitney-Wilcoxon test was performed and FDR-corrected *p* < 0.05 was considered statistically significant.

## Results

### AAV9-mediated transduction efficacy following IP injection as visualized in frozen ring sections

**Figure 1:**
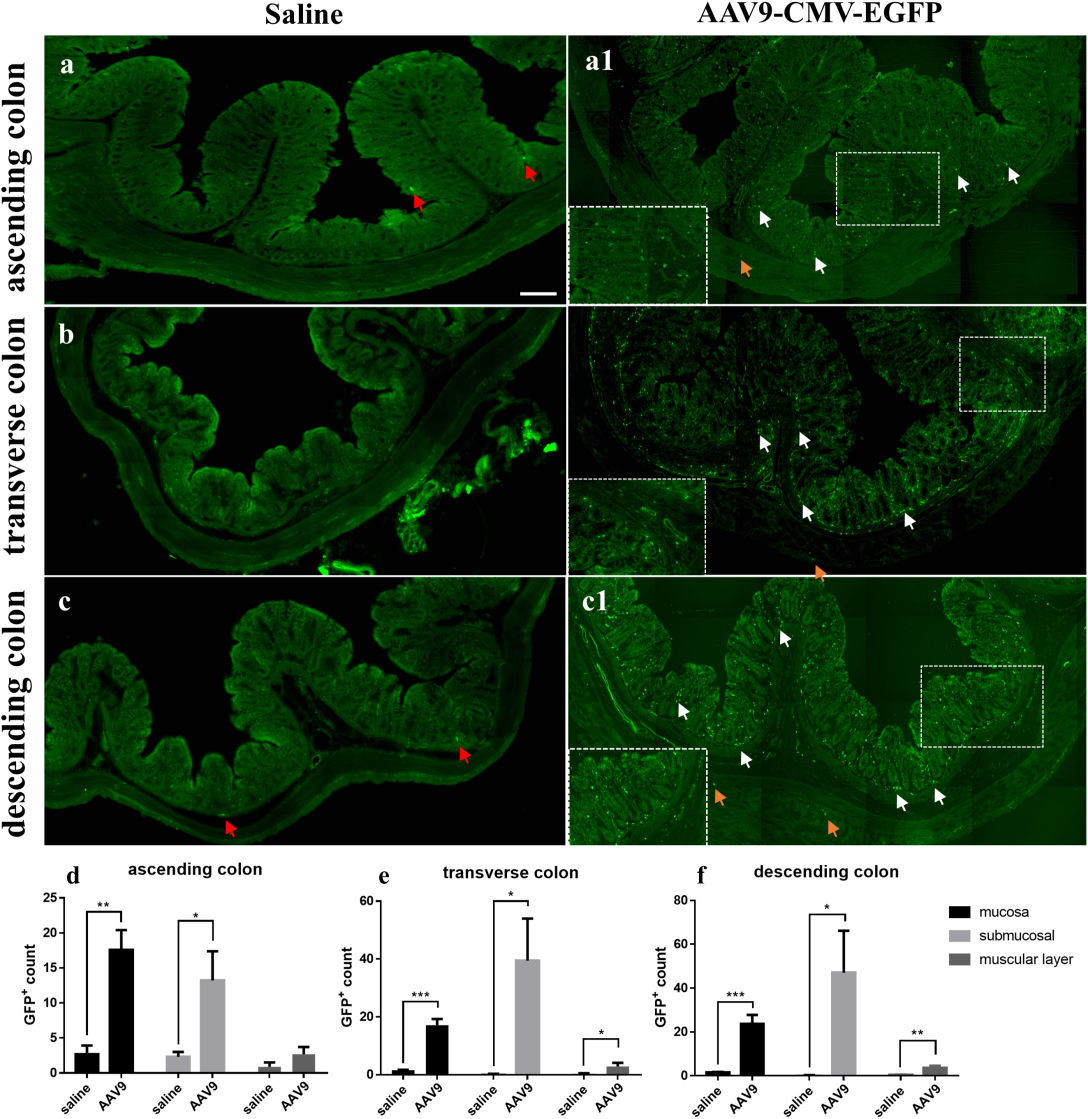
AAV9-mediated transduction efficacy following IP injection as visualized in frozen ring sections. Rats were injected with AAV9 carrying eGFP under the control of a ubiquitous CMV promoter. **(A–C)** The ascending, transverse, and descending colon of the saline group; (**A1, B, C1)** The ascending, transverse, and descending colon of the AAV9 group; **(D–F)** GFP+ cells in the mucosal, submucosal and muscle layers of the ascending, transverse, and descending colon (*n* = 3). The white arrows represent GFP+ cells partially in the mucosa and submucosa, the yellow arrows represent GFP+ cells partially in the muscle layer and the red arrows represent nonspecific fluorescent substances. ^*^p < 0.05; ^**^p < 0.01; ^***^p < 0.01, Scale bar = 200 μm.

Nine rats were injected with AAV9 carrying eGFP under the control of a ubiquitous cytomegalovirus (CMV) promoter; of these, 3 rats were randomly selected for examination and counting. One month after injection, the number of GFP-positive (GFP+) cells in frozen ring sections of each colon segment (the ascending, transverse, and descending segments) was counted and used as an indicator of transduction efficiency. Frozen ring section analysis revealed the following: (1) GFP+ cells were observed in all segments (the ascending, transverse, and descending segments) of the selected colon (**Figures 1A1, B, C1**). (2) GFP+ cells were mainly distributed in the mucosa and submucosa, with less distribution in the muscle layer (**Figures 1D–F**); especially in the mucosa, we can see a large number of scattered GFP+ cells. The white arrows represent GFP+ cells partially in the mucosa and submucosa and the yellow arrows represent GFP+ cells partially in the muscle layer. (3) In the saline group, there was no large number of GFP+ cells. Interestingly, very few substances with green fluorescence could be seen in the saline group (**Figures 1A–C**, red arrow). These substances were similar in size to the morphology and GFP+ cells in the AAV9 group, but they were few in number. The reason why these substances appeared in the saline group may be that the gut itself contains some spontaneous fluorescent substances, especially some microorganisms. In summary, these results indicated that through IP injection, AAV9 was distributed in the mucosa and submucosa of the ascending, transverse, and descending colon.

### Sequencing data analysis

**Figure 2:**
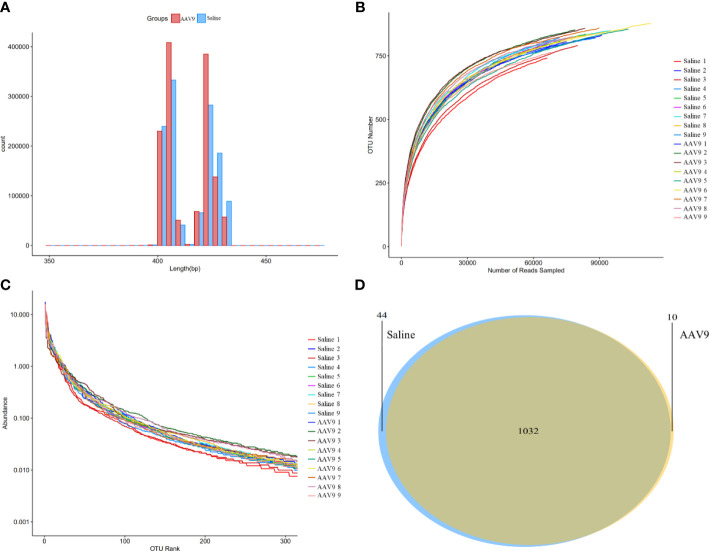
Sequencing data analysis and OTU cluster analysis. **(A)** The length distribution diagram of the valid data. **(B)** The rarefaction curve for each sample. **(C)** The rank-abundance curve for each sample. There was no significant difference between the AAV9 group and the saline group. **(D)** Venn diagram of the two groups. Different groups are represented by different colours, and the numbers in the overlapping parts indicate the number of species in common in the two groups.

Through large-scale sequencing analysis based on the 16S rRNA gene sequence, we analysed the different bacterial communities present after IP injection of AAV9. The original image data files obtained by Illumina HiSeq™ were converted into original sequenced reads by base calling (**Supplemental Table 1**). These sequenced reads were PE sequence data and contained barcode sequences as well as added primers and linker sequences during sequencing. We first removed the primer adapter sequence, merged the PE reads into a sequence according to the overlap relationship between the PE reads, identified and distinguished the samples according to the barcode tag sequence to obtain each sample’s data, and finally performed quality control for each sample’s data to obtain the valid data for each sample (**Figure 2A** and **Supplemental Table 2**).

A certain number of sequences was randomly selected to create a rarefaction curve. The amount of extracted data was on the horizontal axis, and the alpha-diversity index value was on the vertical axis (**Figure 2B**). A rarefaction curve can be used to evaluate sequencing data and is sufficient to represent all bacterial strains present in each sample according to whether the curve is flat. The flatness of each rarefaction curve for the 18 samples showed that we adequately collected the experimental material.

### OTU cluster analysis

The bioinformatics and statistical analyses were performed at the OTU level with a 97% similarity index. The total number of OTUs obtained by clustering the valid data was 1086, and the community composition of every sample was determined at each classification level (domain, phylum, class, order, family and genus).

A rank-abundance curve was used to explain two aspects of sample diversity, that is, the richness and uniformity of the species contained in the samples. The abundance of species is reflected by the length of the curve on the horizontal axis; the further the curve extends on the horizontal axis, the richer the composition of the species. The uniformity of the species composition is reflected by the shape of the curve; the flatter the curve is, the higher the uniformity of the species composition (**Figure 2C**).

Furthermore, a Venn diagram was constructed to count the number of shared and unique OTUs in the samples and to intuitively show the similarity of the OTUs in the samples (**Figure 2D**). The saline and AAV9 groups shared 1032 OTUs. The numbers of unique OTUs that were not common or overlapping in the two groups were 44 in the saline group and 10 in the AAV9 group. These results indicated that the bacterial community structure underwent significant changes following AAV9 IP injection.

### Increased alpha diversity and altered overall microbial composition following AAV9 IP injection

**Figure 3:**
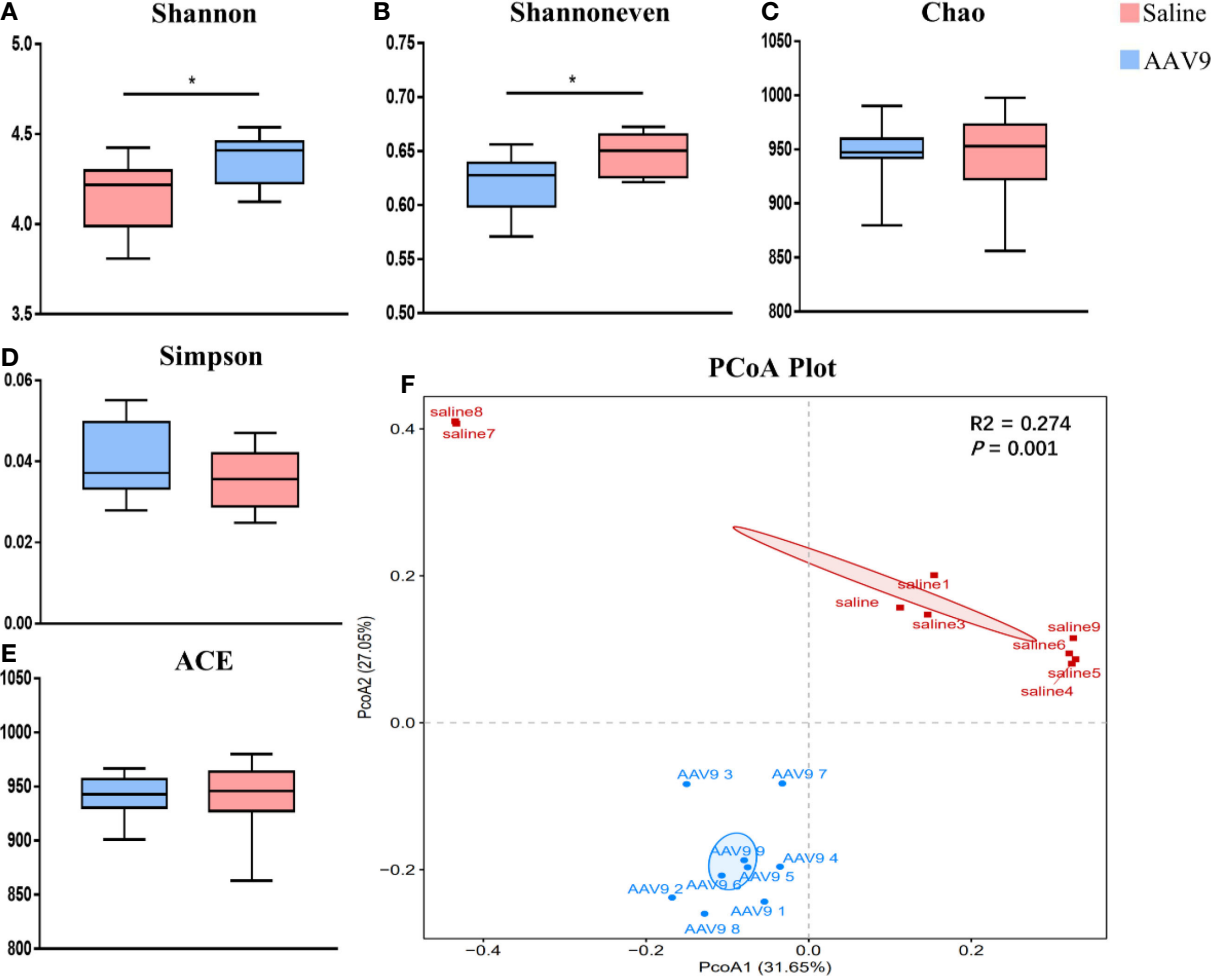
Comparison of the faecal microbiota compositions of the saline and AAV9 groups. **(A)** Shannon, **(B)** Shannon evenness, **(C)** Chao, **(D)** Simpson, **(E)** ACE. Each box plot represents the median, interquartile range, minimum, and maximum values. *P* values were determined using Student’s *t* test. **(F)** PCoA based on Bray–Curtis dissimilarity showed that the overall faecal microbiota composition was different between the saline and AAV9 groups (F. Model: 6.05, *P* = 0.001). ^*^ *p* < 0.05.

To assess the alterations in the alpha diversity and microbial composition after AAV9 IP injection, we performed analyses of community richness (the *Chao* and *ACE* indices), community diversity (the *Shannon* and *Simpson* indices) and community evenness (the *Shannon evenness* index) as well as principal coordinates analysis (PCoA). The AAV9 group showed significantly increased community diversity, as indicated by an increased Simpson index (saline vs. AAV9: 4.161 vs. 4.355, *P* < 0.05), and significantly increased community evenness, as indicated by an increased Shannon evenness index (saline vs. AAV9: 0.6205 vs. 0.6475, *P* < 0.05) (**Figures 3A, B**). The *Chao, Simpson* and *ACE* indices showed no significant differences between groups (**Figures 3C–E**). Importantly, significant compositional differences were also found in the PCoA plot based on Bray–Curtis dissimilarity (PERMANOVA, Bray–Curtis, saline vs. AAV9, *P* = 0.001) (**Figure 3F**). In addition, the distinctive faecal microbial communities associated with AAV9 were evaluated by partial least-squares-latent structure discriminate analysis (PLS-DA) plotting (**Supplemental Figure 1**).

### Specific faecal microbial signature following AAV9 IP injection

**Figure 4:**
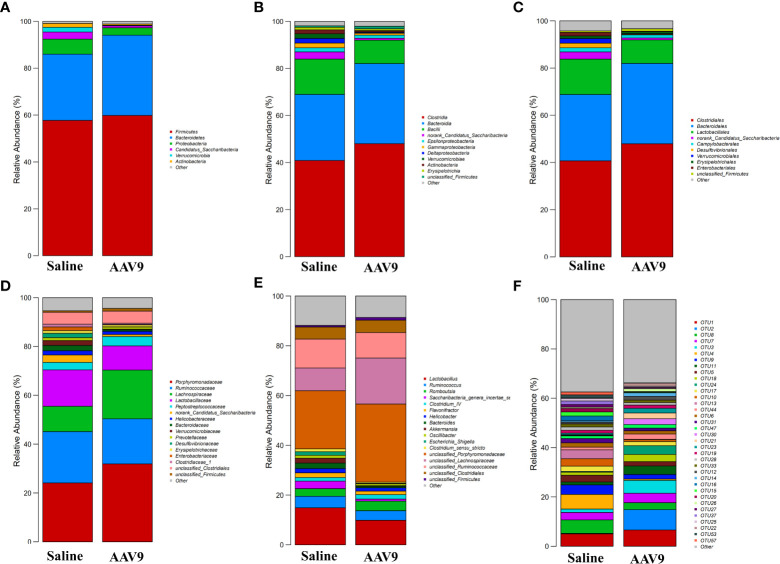
Histogram of the relative abundance of dominant species in the two groups. **(A–F)** Phylum, class, order, family, genus, and OTU levels. The horizontal axis is the corresponding group, and the vertical axis is the relative abundance ratio of species. The colour corresponds to the name of each species at this taxonomic level, and the height of different coloured blocks indicates the relative abundance ratio of different species.

**Figure 5:**
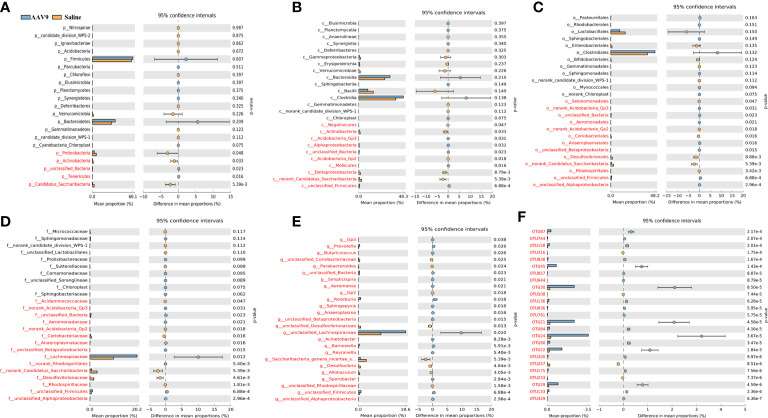
Differential abundance of faecal microbiota in the saline and AAV9 groups. The taxa were altered following AAV9 IP injection in the AAV9 group at the phylum **(A)**, class **(B)**, order **(C)**, family **(D)**, genus **(E)**, and OTU **(F)** levels. Blue and orange represent the AAV9 (n = 9) and saline (n = 9) groups, respectively. In each thumbnail, on the left is the abundance ratio of different taxa in the two groups, in the middle is the percentage difference in species abundance within the 95% confidence interval, and on the right is the p value. *P* < 0.05 indicates a significant difference, marked in red. Notes: At the genus and OTU levels, 25 had the lowest p value.

**Figure 6:**
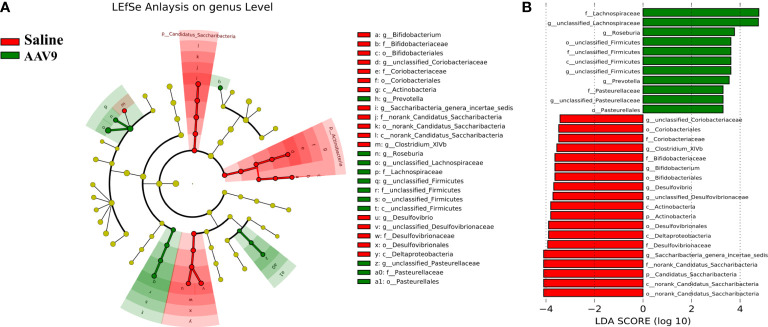
Comparative LEfSe analysis of microbial abundance at the genus level between the two groups. **(A)** LEfSe analysis at the genus level. The green node represents the relatively higher abundance of bacteria at this level in the AAV9 group; the red node represents the higher abundance of bacteria at this level in the saline group. **(B)** Linear discriminant analysis (LDA) score identifying differentiating biomarkers between the two groups with a threshold score of 2.

We examined the abundances of phyla, classes, orders, families, genera, and OTUs in the two groups. The dominant species at each level are shown in a heatmap (**Figure 4**). Next, STAMP was used to compare the species abundances in the two groups (**Figure 5**). In total, 5 phyla, 10 classes, 13 orders, 15 families, 29 genera, and 230 OTUs showed significant differences between the two groups. At the phylum level, 5 phyla showed significant differences: the relative abundances of *Candidatus_Saccharibacteria*, *Actinobacteria* and *Proteobacteria* were decreased (saline vs. AAV9: 3.13% vs. 0.81%, 1.69% vs. 0.53%, and 6.23% vs. 3.17%, respectively), and the relative abundances of *Tenericutes* and unclassified_*Bacteria* were increased (saline vs. AAV9: 0.004% vs. 0.13% and 0.42% vs. 0.54%) in the AAV9 group (**Figure 5A**). Similar significant alterations in the microbiome in the AAV9 group were found at the corresponding class, order, family, genus, and OTU levels (**Figures 5B–F**). Among the results of the STAMP, some microorganisms do not have names at the genus level, and we label them as unclassified. Among the microorganisms with specific names at the genus level, ***Saccharibacteria_genera_incertae_sedis***, ***Desulfovibrio*** and ***Allobaculum*** are of interest because AAV9 significantly reduces their abundance compared to the saline group. In contrast, the abundance of ***Prevotella***, ***Roseburia***, and unclassified ***Lachnospiraceae*** increased significantly in group AAV9. Furthermore, we confirmed the distinguishing microorganisms between the two groups at the genus level by LEfSe analysis (Zhang et al., 2013) (**Figure 6**). LEfSe analysis can identify the factors that are most likely to explain different categories and can identify biomarkers with significant differences. The results showed that different groups at different taxonomic levels can be distinguished across the sites. In the LEfSe analysis, *unclassified* ***Lachnospiraceae*** at the genus level obtained the highest LDA score. In second place at the genus level is ***Roseburia***. Next to note are *unclassified Firmicutes*, ***Prevotella*** and *unclassified Pasteurellaceae.* Regrettably, no specific microorganisms of Firmicutes and Pasteurellaceae were found at the genus level. This will be determined by metagenomic sequencing in our future studies.

### PICRUSt prediction of the metabolic functions of rat intestinal microbiota after AAV9 IP injection

**Figure 7:**
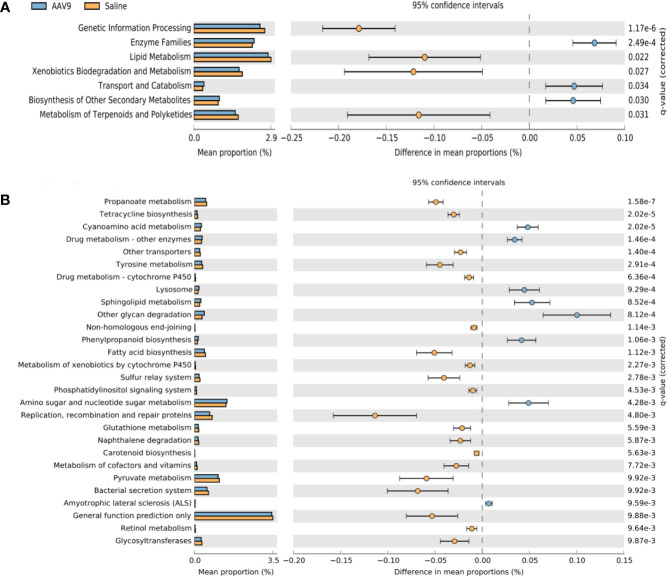
Prediction of PICRUSt function of rat faecal microbiota after AAV9 IP injection. STAMP software was used to perform statistical calculations and analyses and to visualize different KEGG pathway levels. **(A)** KEGG level 2 (*P* < 0.05); **(B)** KEGG level 3 (*P* < 0.01).

Phylogenetic investigation of communities by reconstruction of unobserved states (PICRUSt) was used to compare the obtained 16S rRNA gene sequencing data with the data in a microbial reference genome database with known metabolic functions to predict the metabolic functions of the bacteria and archaea present in the samples (Langille et al., 2013). The gene sequence measured by 16S rRNA was “mapped” to the known Kyoto Encyclopedia of Genes and Genomes (KEGG) function profile database to predict the metabolic function of the microbiota, and STAMP software was used for statistical calculations and data analysis. At level 2, compared with the saline group, the AAV9 group had significantly reduced pathway function genes related to “genetic information processing”, “lipid metabolism”, “metabolism of terpenoids and polyketides” and “xenobiotic biodegradation and metabolism”. In contrast, the AAV9 group had significantly increased pathway function genes related to “enzyme families”, “biosynthesis of other secondary metabolites” and “transport and catabolism” (**Figure 7A**). In level 3, a total of 37 pathways were found to have undergone significant changes. The figure shows a pathway with *P* < 0.01 (**Figure 7B**).

### AAV9 can up-regulate the abundance of anaerobic fecal microbiota

**Figure 8:**
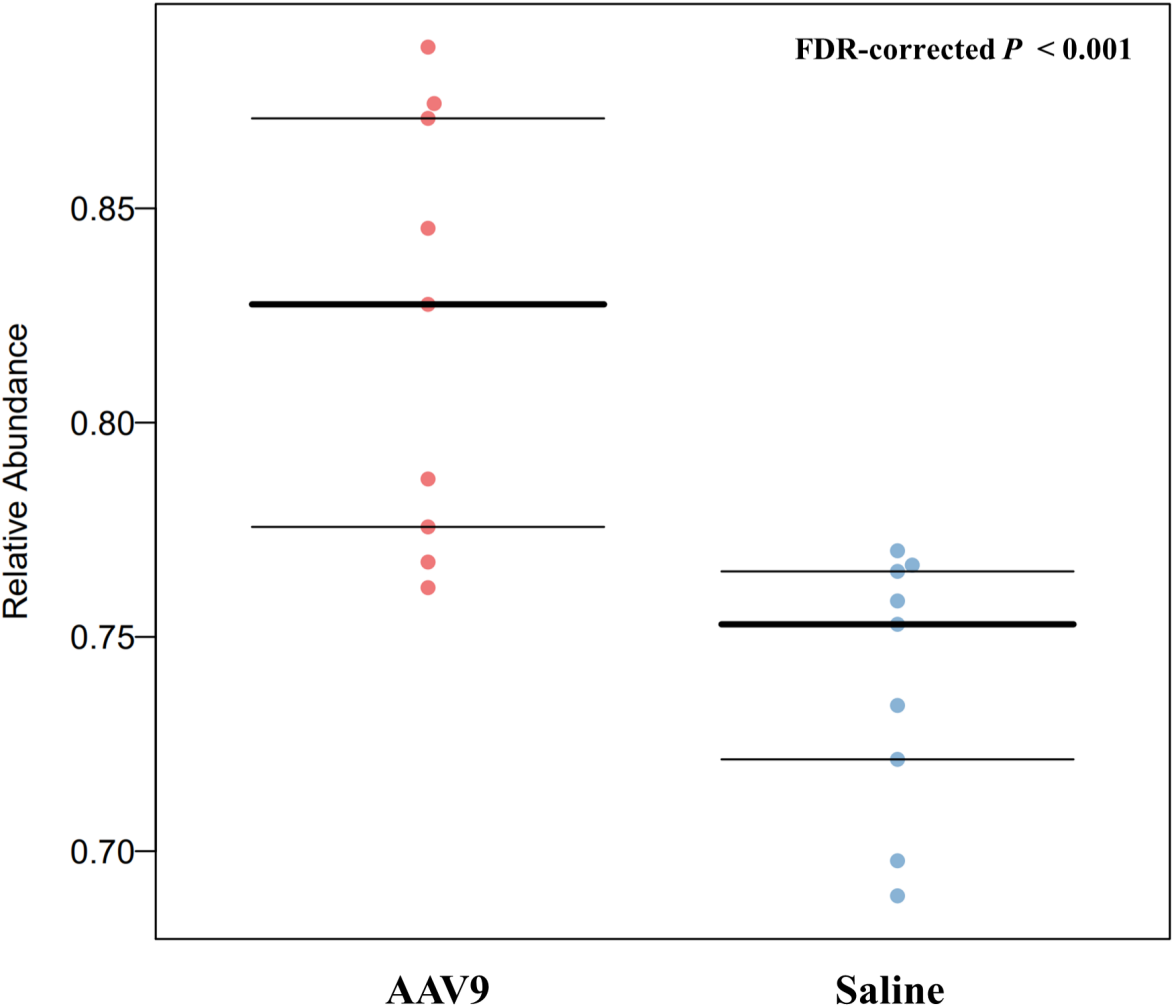
AAV9 can upregulate the abundance of anaerobic faecal microbiota. The horizontal axis is the group name, and the vertical axis is the relative abundance. The anaerobic microbiota was significantly different between the two groups. AAV9 can significantly upregulate the relative abundance of anaerobic microbiota (FDR-corrected *P* < 0.001, Mann‐Whitney-Wilcoxon).

BugBase can determines high-level phenotypes present in microbiome samples (https://bugbase.cs.umn.edu/). Of the seven high-level phenotypes that BugBase could predict, only anaerobic microbiota was statistically significantly different between the two groups. AAV9 can significantly upregulate the relative abundance of anaerobic microbiota (FDR-corrected *P* < 0.001, **Figure 8**), and has no effect on the remaining phenotypes (**Supplemental Figure 2**).

## Discussion

Through AAV9 IP injection, we observed a large number of GFP+ cells in the mucosal layer and submucosal layer of the ascending colon, transverse colon and descending colon. These results show that AAV9 can successfully infect these sites. However, this method of observation is limited because the mucosal layer occupies a relatively large portion of each slice, while the submucosal layer and the muscle layer occupy a relatively small portion. In this study, we did not go further to explore what cells are being transduced, as there have been many studies that have investigated by immunohistochemistry and immunofluorescence (Sferra et al., 1997; Polyak et al., 2012; Gombash et al., 2014; Benskey et al., 2015; Buckinx and Timmermans, 2016; Buckinx et al., 2016; Gombash et al., 2017). Moreover, the IP injection route was chosen to deliver AAV9 because it is convenient to perform and could lead to efficient transduction of the colonic mucosa. In fact, because of protective extracellular barriers such as tight junctions and glycocalyx, transferring genes to the intestinal wall is quite difficult (Wirtz and Neurath, 2003). Currently, the routes of viral administration mainly include enema or intracolonic administration (Wirtz and Neurath, 2003; Hao et al., 2012), oral feeding (During et al., 1998), intravenous injection (Villiger et al., 2018), arterial injection (Chang et al., 2003), local intestinal tissue injection (Hao et al., 2012), and IP injection (Ikezoe et al., 2021). These methods have been used in different studies, and the results have varied. Studies have shown that rectal enema can induce adenoviral particles to efficiently transduce the colon mucosa. However, taking into account the high turnover rate of resorptive enterocytes, adenovirus-mediated expression is transient and decreases sharply after 2-3 days (Wirtz et al., 1999; Wirtz and Neurath, 2003). Injecting of the virus directly into the wall of the descending colon in adult rats covered an area of ~47 mm^2^ (Benskey et al., 2015). Superior mesenteric artery injection might be a more efficient route for transferring AAV to the small intestine and colonic epithelial cells *in vivo* (Polyak et al., 2008). All of these routes have advantages and disadvantages. Intravenous injection, arterial injection and IP injection appear to be more efficient in transferring virions to the intestine. Unfortunately, in theory, given the rapid flow of blood, these methods inevitably carry viral vectors to other parts of the body. Among these methods, IP injection is the most convenient method and our experimental results suggest that it can successfully deliver AAV to the intestinal mucosa and cause changes in the gut microbiota.

Studies have reported on the time course of AAV-mediated gene expression. In a study that delivered ghrelin to the heart using an AAV9 carrier, ghrelin expression was observed after 7 days (*P* < 0.01) of injection, was more pronounced after 21 days (P < 0.001), and maintained high levels for at least three months (Ruozi et al., 2015). In addition, in the literature, 30 days or 60 days are often used as the test time points to detect the AAV transduction efficiency (Gombash et al., 2014). For these reasons, we chose 30 days to euthanize rats after injection and collect tissues. In addition, the autofluorescence of microorganisms had to be taken into account when counting the number of GFP+ cells. In 2017, Shu-hong Tian et al (TIAN Shu-hong and XIAO Min, 2017) used Kinetics IVIS to study the distribution of autofluorescent microorganisms in the rat intestine. The results showed that in the 8-week-old rat intestine, autofluorescent material was mainly distributed in the ileum, with small amounts in the jejunum, rectum, stomach and caecum (TIAN Shu-hong and XIAO Min, 2017). Analogously, this phenomenon of spontaneous fluorescence was also encountered in our statistics. Specifically, very few substances with green fluorescence were observed in the saline group (**Figures 1A–C**). These substances were similar in size to the morphology and GFP+ cells in AAV9 group, but they were few in number. The reason why these substances appeared in the saline group may be that the gut itself contains some spontaneous fluorescent substances, especially some microorganisms. In the process of statistical data, as we describe: In frozen slices, three 500 µm^2^ areas were randomly selected for counting the number of GFP+ cells in the mucosa, and it is inevitable that a small number of nonspecific cells with spontaneous fluorescence were counted in the saline group. This may explain why the saline group had GFP+ counts in the histogram showen in **Figure 1**.

It is generally accepted that the interaction between the host and the microbiome plays a key role in various diseases, but the mechanism by which the host regulates the microbial community remains unclear. It has been reported that gut miRNAs exist in faeces and can regulate the gut microbiome (Liu et al., 2016; He et al., 2022). The ability of AAV9 to infect the colonic mucosa has been demonstrated previously and in this study. However, extensive research is still needed to reveal how AAV9, which infects mucosal epithelial cells, induces changes in the regulation of intestinal microbiota by intestinal mucosal epithelial cells. Yasuda et al (Yasuda et al., 2015) studied the microbiota of the distal jejunum of rhesus monkeys and concluded that the structure of the faecal microbiota was closely related to the colon community; but only moderately related to the microbial community in the small intestine. In our study, we focused on the effect of IP injection of AAV on the faecal microbiota of rats. The faecal microbiota was most associated with the colon rather than the small intestine. Therefore, we focused on examining the infection of colon tissue by AAV.

In addition to these conclusions, we further revealed that AAV9 IP injection can increase the alpha diversity of faecal microorganisms, alter the overall microbial composition and change the metabolic function of the microbiota. AAV gene therapy has been approved by the FDA for the treatment of the early childhood blindness disease Leber congenital amaurosis (Kumaran et al., 2018) and spinal muscular atrophy (SMA) (Hoy, 2019), and almost all gene editing work applied in the clinic relies on AAV vectors to deliver a genetic payload (Dunbar et al., 2018; High and Roncarolo, 2019), but long-term longitudinal clinical research data and extensive basic experiments are still needed for further research on safety.

When we use viruses to treat human genetic diseases, we must not forget that the gut microbiota that lives with us from birth to death (Van Treuren and Dodd, 2020; Gu et al., 2021). Gut microorganisms play a key role in maintaining health dysbiosis and induce inflammation and metabolic dysfunctions that are associated with metabolic, GI, and neurological diseases; GI tract cancers; and atherosclerotic cardiovascular diseases (Zununi Vahed et al., 2018; Yu et al., 2021). In a previous study, “Long-Term Follow-Up of the First in Human Intravascular Delivery of AAV for Gene Transfer” (George et al., 2020), a haemophilia B patient treated with AAV2-hFIX16 died following myocardial infarction related to underlying atherosclerosis; on the basis of the data, due to the sample size, we cannot conclude whether the incident was causally related to the AAV treatment. However, what we cannot ignore is that the existing evidence shows that the intestinal microbiota is most likely to affect the occurrence and development of acute myocardial infarction through short-chain fatty acid pathways (Dannenberg et al., 2020; Zhou et al., 2020; Han et al., 2021; Li et al., 2021). Notably, the intestinal microbiota is regulated by the host’s intestinal mucosal epithelial cells, and IP injection of AAV can lead to AAV infection of intestinal mucosal epithelial cells. Whether this lasting change in the diversity and composition of the intestinal microbiota would lead to pathological changes in other human systems is still unknown; therefore, we need to further explore the safety of AAV and its impact on the ecology of the entire body.

In this study, AAV9 IP injection led to changes in the composition of the dominant microbiota at all levels (the phylum, class, order, family and genus levels). The introduction of AAV9 led to an increased abundance of *Prevotella*, *Roseburia and unclassified Lachnospiraceae.* Whether *Prevotella* is beneficial or harmful to human health remains a major controversy, and research evidence is often controversial (Ley, 2016; Claus, 2019). The beneficial aspects of a gut microbiota rich in *Prevotella* are that it helps to reduce weight (Hjorth et al., 2018), lower cholesterol levels (Eriksen et al., 2020), and inhibit the bifidogenic effect (Chung et al., 2020). Other studies have reported that members of the genus *Prevotella* are related to a variety of diseases, including inflammatory autoimmune diseases (Scher et al., 2013) and opportunistic infections (Zhao-Fleming et al., 2018). Interestingly, the introduction of AAV9 increased the abundance of *Roseburia*, which could improve intestinal biodiversity, increase glucose tolerance, help weight loss, and rejuvenate colon cells (Tamanai-Shacoori et al., 2017). In addition, *Roseburia*, a high-producing butyric acid bacterium, may play an important role in controlling the inflammatory process, especially the intestinal inflammatory process (Estevinho et al., 2020). Similar to *Prevotella*, the role of human gut *Lachnospiraceae* is still controversial (Vacca et al., 2020). *Blautia*, a genus of Firmicutes in the family *Lachnospiraceae*, is associated with a reduced risk of lethal acute graft-versus-host disease and improved overall survival (Jenq et al., 2015). However, other studies have reported that the gut microbiota of patients with nonalcoholic fatty liver disease is rich in *Lachnospiraceae*, especially *Blautia* and *Lachnospiraceae incertae sedis(* Shen et al., 2017 *)*, and there is a close relationship between *Lachnospiraceae* and impaired glucose metabolism (Hill et al., 2016; Wang et al., 2021).

In addition to microbiomes with increased abundance, AAV9 could also decrease the abundance of some microorganisms. There are three notable taxa: *Allobaculum, Desulfovibrio* and *Saccharibacteria_genera_incertae_sedis*. Similar to *Roseburia*, *Allobaculum* has been reported to have many health benefits. For example, *Allobaculum* can improve metabolic syndrome, prevent the inflammation caused by dextran sulfate, and protect intestinal barrier function by producing short-chain fatty acids (Ma et al., 2020; Yao et al., 2021). *Desulfovibrio* can use sulfate instead of oxygen and produce hydrogen sulfide (H_2_S). Most studies have shown that endogenous H_2_S is harmful to human health. The substances (H_2_S, lipopolysaccharide and several strains synthesize magnetite) derived from *Desulfovibrio* likely take part in the pathogenesis of Parkinson’s disease (Murros et al., 2021). There is also evidence that the abundance of the *Desulfovibrio* subspecies is increased in ulcerative colitis (Rowan et al., 2010). For *Saccharibacteria_genera_incertae_sedis*, studies have shown that a high-fat diet causes an increase in Wistar rats (Jin and Zhang, 2020), and an increased abundance of *Saccharibacteria_genera_incertae_sedis* was also found in patients with diabetes (Zhang et al., 2020). In general, IP injection of AAV9 not only increased the alpha diversity of the faecal microbiota but also seemed to increase the abundance of beneficial bacteria. Whether these changes in faecal microbiota will have a beneficial effect on the body still needs further research.

Using PICRUSt analysis, based on the results of high-throughput sequencing, we predicted and analysed the changes in the metabolic pathways of intestinal microbiota caused by IP injection of AAV9. The introduction of AAV9 induced decreased abundances of “acetyl-CoA carboxylase biotin carboxyl carrier protein”, “acetyl-CoA carboxylase carboxyl transferase subunit alpha”, and “pyruvate dehydrogenase E1 component subunit beta”. Acetyl-CoA carboxylase carboxylases (ACCases) are necessary for the survival and reproduction of bacteria and are important regulatory enzymes for bacterial growth and fatty acid metabolism (Payne et al., 2007). The introduction of AAV9 may induce a reduction in the synthesis of ACCases at level 2 accompanied by a downregulation of lipid metabolism. Almost all microorganisms need to synthesize fatty acids to build cell membranes or participate in basic metabolism. ACCases as targets are easy to screen with antibiotics with a broad spectrum of resistance. A decline in ACCases indicates that the bacteria have been inhibited. We cannot explain why the introduction of AAV9 leads to the inhibition of intestinal bacteria, but a complex interaction of AAV, intestinal mucosal epithelial cells and intestinal microbiota, as well as the immune system, may be involved.

To our knowledge, this is the first research report on the changes in intestinal microbiota caused by AAV9. Previous studies on viruses and intestinal microbiota have focused on papillomavirus (Mei et al., 2022), rotavirus or norovirus (Peña-Gil et al., 2021), hepatitis C virus (Honda et al., 2021) and so on. AAV is an ideal candidate for gene therapy due to its low insertion rate and low immunogenicity. Either intravenous injection or superior mesenteric artery injection of AAV can successfully induce transduction of the ENS in the stomach, small intestine and large intestine (Gombash et al., 2014; Buckinx and Timmermans, 2016; Buckinx et al., 2016; Gombash et al., 2017). Studies have also shown that IP injection of AAV can lead to successful transduction of adipose (Queen et al., 2021) and heart (Rotundo et al., 2011; Schlesinger-Laufer et al., 2020) tissue. AAV IP injection is simple and flexible. However, its impact on the intestinal microecology, which is closely related to human health, is still unknown. Our research confirms that AAV9 has an effect on faecal microbiota, and further studies are needed to confirm whether the changes in faecal microbiota can recover on their own and to determine the effects on human health. Moreover, when we use AAV in gene therapy, we should note that although AAV can be used to deliver genes, AAV itself can lead to changes in intestinal microecology, especially when AAV is used in the treatment of digestive system diseases.

## Conclusion

IP injection of AAV9 can successfully induce the transduction of the colonic mucosa and submucosa and alter the diversity and composition of the faecal microbiota in rats. These results call for us to consider the effect of AAV on the gut microbiome when using AAV for gene therapy.

## Data availability statement

The datasets presented in this study can be found in online repositories. The names of the repository/repositories and accession number(s) can be found below: https://www.ncbi.nlm.nih.gov/Traces/study/?acc=PRJNA798156.

## Ethics statement

The animal study was reviewed and approved by Animal Care and Use Committee of Air Force Medical University (permit number IACUC-20200503).

## Author contributions

J-JW, Y-QL and TL designed the study and approved the final version of the manuscript. L-TM, J-XL and YB performed the experiments and wrote the manuscript. M-JS, Z-ZZ and F-FW performed the data analysis. JC, X-BM and JZ participated in part of 16S rRNA gene amplicon sequencing. All authors contributed to the article and approved the submitted version.

## Supplementary material

Supplementary Figure 1 PLS-DA plotting. Points of different colours or shapes represent sample groups under different conditions. The scales on the horizontal and vertical axes are relative distances and have no practical meaning. Blue represents the AAV9 group; red represents the saline group. The two groups were separated in the direction of the PLSDA1 axis, verifying that this factor has a higher possibility of affecting the composition of the sample (OTU level).

Supplementary Figure 2 Effects of AAV9 on the remaining phenotypes in faecal microbiota by BugBase analysis. AAV9 can significantly upregulate the relative abundance of anaerobic microbiota, and has no effect on the remaining phenotypes: gram-positive, gram-negative, biofilm forming, pathogenic, mobile element containing, oxygen utilizing (including aerobic and facultatively anaerobic) and oxidative stress tolerant.

Supplementary Table 1 The original sequenced reads of the 16S rRNA gene of bacterial communities in all samples. Column 1 lists the sample names. Columns 2 and 3 list the valid data and base number, respectively, of each sample after performing quality control. Columns 4, 5 and 6 list the average read length, minimum read length and maximum read length, respectively.

Supplementary Table 2 Valid sequencing analysis data of the 16S rRNA gene of bacterial communities in all samples. Column 1 lists the sample names. Columns 2 and 3 list the valid data and base number, respectively, of each sample after performing quality control. Columns 4, 5 and 6 list the average read length, minimum read length and maximum read length, respectively.
