# Supplementary figures and images for: Adeno-associated virus vector intraperitoneal injection induces colonic mucosa and submucosa transduction and alters the diversity and composition of the faecal microbiota in rats
Source: Front Cell Infect Microbiol. 2022 Dec 22;12:1028380. doi: 10.3389/fcimb.2022.1028380 (PMC9813966; doi:10.3389/fcimb.2022.1028380)

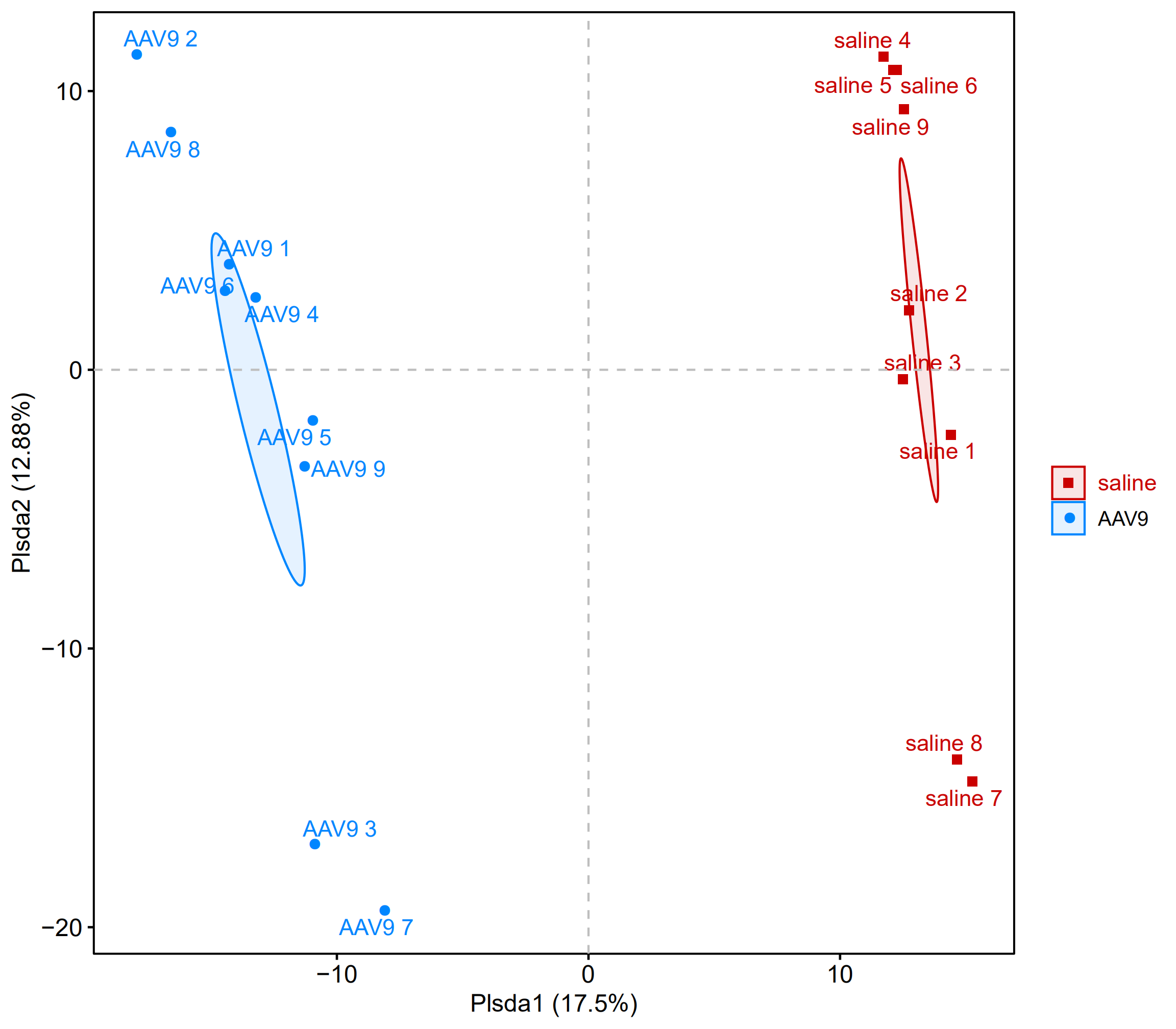

Supplement: Supplementary Figure 1 — PLS-DA plotting. Points of different colours or shapes represent sample groups under different conditions. The scales on the horizontal and vertical axes are relative distances and have no practical meaning. Blue represents the AAV9 group; red represents the saline group. The two groups were separated in the direction of the PLSDA1 axis, verifying that this factor has a higher possibility of affecting the composition of the sample (OTU level). [file Image_1.tif]

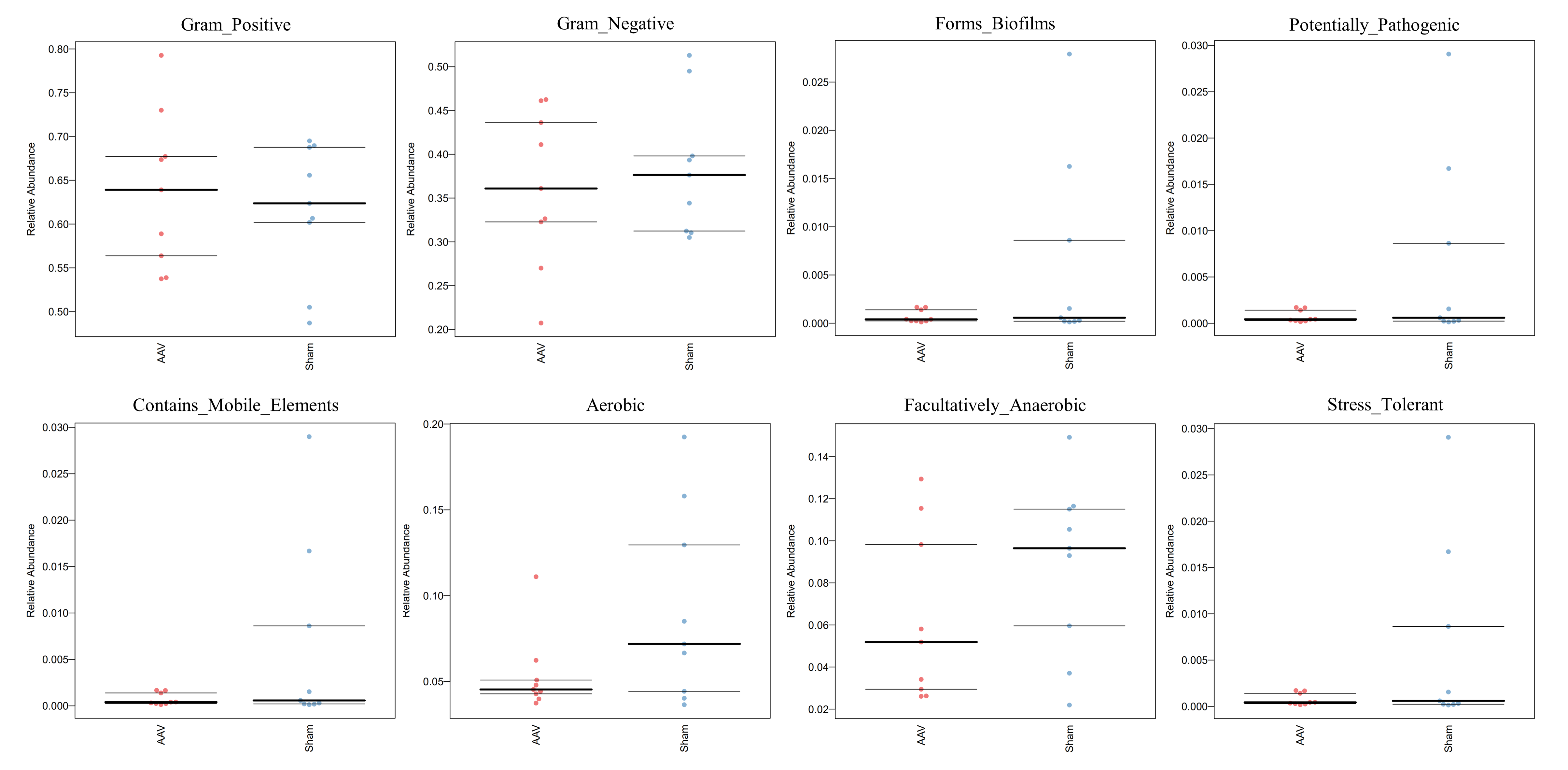

Supplement: Supplementary Figure 2 — Effects of AAV9 on the remaining phenotypes in faecal microbiota by BugBase analysis. AAV9 can significantly upregulate the relative abundance of anaerobic microbiota, and has no effect on the remaining phenotypes: gram-positive, gram-negative, biofilm forming, pathogenic, mobile element containing, oxygen utilizing (including aerobic and facultatively anaerobic) and oxidative stress tolerant. [file Image_2.tif]
