# Supplementary material for: Adeno-associated virus vector intraperitoneal injection induces colonic mucosa and submucosa transduction and alters the diversity and composition of the faecal microbiota in rats
Source: Front Cell Infect Microbiol. 2022 Dec 22;12:1028380. doi: 10.3389/fcimb.2022.1028380 (PMC9813966; doi:10.3389/fcimb.2022.1028380)
Supplement: Supplementary Table 1 — The original sequenced reads of the 16S rRNA gene of bacterial communities in all samples. Column 1 lists the sample names. Columns 2 and 3 list the valid data and base number, respectively, of each sample after performing quality control. Columns 4, 5 and 6 list the average read length, minimum read length and maximum read length, respectively. [file Table_1.pdf]

Sample	SeqNum	BaseNum	MeanLen	MinLen	MaxLen
Saline 1	108493	49163629	453.15	60	506
Saline 2	150556	68085204	452.23	59	509
Saline 3	129082	58120773	450.26	45	506
Saline 4	130895	59420910	453.96	62	513
Saline 5	141643	64122651	452.71	56	511
Saline 6	132839	60020149	451.83	60	511
Saline 7	155953	69823622	447.72	55	507
Saline 8	160065	72126504	450.61	63	512
Saline 9	142230	63978377	449.82	46	509
AAV9 1	133943	60689235	453.1	59	510
AAV9 2	149279	67183076	450.05	65	510
AAV9 3	145370	65249553	448.85	62	505
AAV9 4	128627	57985935	450.81	61	507
AAV9 5	173480	78731643	453.84	60	506
AAV9 6	212522	95904651	451.27	59	509
AAV9 7	151988	68363417	449.79	46	503
AAV9 8	141313	63734193	451.01	63	513
AAV9 9	115782	51569630	445.4	46	511
