# Supplementary material for: Adeno-associated virus vector intraperitoneal injection induces colonic mucosa and submucosa transduction and alters the diversity and composition of the faecal microbiota in rats
Source: Front Cell Infect Microbiol. 2022 Dec 22;12:1028380. doi: 10.3389/fcimb.2022.1028380 (PMC9813966; doi:10.3389/fcimb.2022.1028380)
Supplement: Supplementary Table 2 — Valid sequencing analysis data of the 16S rRNA gene of bacterial communities in all samples. Column 1 lists the sample names. Columns 2 and 3 list the valid data and base number, respectively, of each sample after performing quality control. Columns 4, 5 and 6 list the average read length, minimum read length and maximum read length, respectively. [file Table_2.pdf]

Sample	SeqNum	BaseNum	MeanLen	MinLen	MaxLen
Saline 1	107235	44856148	418.3	354	466
Saline 2	149519	62176246	415.84	352	469
Saline 3	126721	52810295	416.74	351	468
Saline 4	129887	54255571	417.71	353	475
Saline 5	140908	58595354	415.84	358	473
Saline 6	131954	54769098	415.06	354	467
Saline 7	155455	63809038	410.47	351	466
Saline 8	158901	65772657	413.92	352	474
Saline 9	140173	58269295	415.7	353	468
AAV9 1	133569	55539169	415.81	351	472
AAV9 2	148933	61440219	412.54	351	472
AAV9 3	145143	59682383	411.2	351	464
AAV9 4	126910	52925865	417.03	354	466
AAV9 5	172895	72035016	416.64	355	466
AAV9 6	210581	87493770	415.49	354	471
AAV9 7	150373	62295837	414.28	354	461
AAV9 8	140815	58268192	413.79	351	475
AAV9 9	112440	46669747	415.06	356	473
